# Short-Term Efficacy and Safety of Hemodialysis Using Super High-Flux Dialyzers Compared With Online Hemodiafiltration: An Exploratory Cross-Sectional Study in Vietnam

**DOI:** 10.7759/cureus.103997

**Published:** 2026-02-20

**Authors:** Thanh-Cong Nguyen, Hong-Vu Le Thi, Phu Quoc Nguyen, Hoai-Vy Nguyen Thi, Ngoc Tran Le Nguyen, Van Song Tran

**Affiliations:** 1 Department of Nephrology and Transplant Immunology, People's Hospital 115, Ho Chi Minh City, VNM; 2 International Ph.D. Program in Medicine, Taipei Medical University, Taipei, TWN

**Keywords:** hemodialysis, interleukin-6, middle molecules, online hemodiafiltration, super high-flux dialyzer, vietnam, β₂-microglobulin

## Abstract

Background

Online hemodiafiltration (OL-HDF) improves middle-molecule clearance but requires ultrapure water systems and specialized infrastructure, limiting implementation in many low- and middle-income countries. Super high-flux (SHF) dialyzers may enhance middle-molecule removal using standard hemodialysis platforms. This exploratory, single-session study evaluated the short-term biochemical efficacy and safety of SHF hemodialysis (SHF-HD) compared with post-dilution OL-HDF in a Vietnamese tertiary center.

Methods

In this cross-sectional pilot study, 30 maintenance hemodialysis patients (15 SHF-HD; 15 OL-HDF) were evaluated during a single 240-minute session. Pre- and post-dialysis concentrations of urea, β₂-microglobulin, parathyroid hormone, interleukin-6, leptin, albumin, and C-reactive protein (CRP) were measured. Reduction ratios were hematocrit-corrected. This study was not powered for non-inferiority or equivalence testing.

Results

Baseline characteristics were comparable, although blood flow rate (Qb) was higher in the OL-HDF group (270 ± 22 vs. 240 ± 24 mL/min; p = 0.008). No statistically significant differences were detected in reduction ratios of β₂-microglobulin (65.05% vs. 68.20%, p = 0.561), parathyroid hormone (66.32% vs. 63.57%, p = 0.803), interleukin-6 (23.62% vs. 21.20%, p = 0.213), or leptin (41.08% vs. 36.36%, p = 0.678). Serum albumin changes were minimal in both groups (−0.40 ± 0.68 vs. −0.44 ± 1.32 g/L, p = 0.911), and CRP remained stable. No intradialytic adverse events occurred.

Conclusions

In this exploratory Vietnamese pilot study, SHF-HD demonstrated similar short-term biochemical performance to OL-HDF during a single dialysis session, without acute safety concerns. Given the small sample size, non-randomized allocation, moderate convective volume, and absence of direct albumin quantification, these findings should be interpreted cautiously. Larger, prospective studies are required to determine long-term clinical implications.

## Introduction

Hemodialysis (HD) is the most commonly used form of kidney replacement therapy worldwide. While conventional adequacy assessment has focused on the clearance of small solutes, it is now recognized that many uremic toxins relevant to long-term outcomes are middle or protein-bound molecules, including β₂-microglobulin, inflammatory cytokines, and advanced glycation end products [1]. These solutes are poorly removed by standard high-flux membranes, leading international guidelines to broaden the concept of dialysis adequacy beyond urea kinetics alone [2].

Online hemodiafiltration (OL-HDF) was developed to improve the removal of middle molecules through convective transport. Large randomized trials and pooled analyses, including the CONVINCE trial [3], the ESHOL (Estudio de Supervivencia de Hemodiafiltración On-Line) study [4], and individual-patient meta-analyses [5,6], have shown improved overall and cardiovascular survival with high-volume OL-HDF compared with high-flux HD. These benefits appear dependent on achieving sufficient convection volumes, as emphasized by the 2021 European Renal Association (ERA) Dialysis Working Group consensus statement [7]. However, OL-HDF requires ultrapure water systems, online substitution monitoring, and strict quality control, which limits its routine use in many low- and middle-income countries [8].

Super high-flux (SHF) dialyzers have been introduced to enhance middle-molecule removal within a purely diffusive HD framework. By modifying membrane pore structure and fiber design, SHF dialyzers increase permeability to larger solutes while maintaining acceptable albumin retention. Randomized studies from Thailand reported that SHF-HD achieves short-term clearance of middle-molecule and protein-bound toxins comparable to post-dilution high-volume OL-HDF [9-11]. Observational studies and registry data from Japan suggest similar survival outcomes between SHF-HD and OL-HDF when albumin leakage is comparable, with improved survival observed in patients treated with type V (super high-flux) dialyzers [12,13]. Comparable findings with medium cut-off membranes support the concept that enhanced diffusive clearance may partly substitute for convective strategies [14-16]. In Vietnam, Nguyen and colleagues combined hemoperfusion with conventional HD and reported a reduction in cardiovascular mortality in a pilot study [17]. Safety considerations remain important. Available data indicate that SHF-HD is well tolerated, without increased intradialytic hypotension, and short-term studies have shown stable serum albumin levels despite increased albumin loss into dialysate [10].

Vietnam’s dialysis system reflects these global contrasts. Maintenance HD has expanded quickly across the country, yet the use of OL-HDF remains modest. Cost constraints, machine limitations, and the demanding requirements for water quality control all contribute to this slow uptake, leaving many centers reliant on conventional high-flux HD despite interest in more advanced modalities [8,18-20]. Despite growing international experience, SHF-HD has not been systematically evaluated in Vietnamese patients, nor has it been directly compared with OL-HDF in this setting. Differences in patient characteristics, diet, and treatment practices may influence both efficacy and safety. This exploratory, cross-sectional pilot study aimed to evaluate the short-term biochemical efficacy and safety of SHF-HD during a single dialysis session in Vietnamese maintenance HD patients and to compare reduction ratios and immediate safety parameters with post-dilution OL-HDF.

This article was previously posted to the Research Square preprint server on December 16, 2025. The findings of this study were presented at the Fifth National Dialysis Conference (2025) of the Vietnam Dialysis Association.

## Materials and methods

Study design 

This cross-sectional study was conducted at the Dialysis Unit of People’s Hospital 115, Ho Chi Minh City, Vietnam, between July and September 2025. The study was carried out under real-world clinical conditions at one of the largest tertiary renal centers in southern Vietnam, which manages a high-volume population of patients with end-stage kidney disease (ESKD). 

All participants received verbal and written explanations and provided written informed consent before enrollment. Ethical conduct followed the principles of the Declaration of Helsinki. The protocol was approved by the Institutional Review Board of People’s Hospital 115 (approval number: 2497/QĐ-BVND115; registration number: CS/15/25/46). This study was not registered on ClinicalTrials.gov because it was not an interventional trial. This investigation should be considered a pilot exploratory study conducted within a fixed timeframe and defined dialysis population.

Study population

Eligibility Criteria

Inclusion criteria were (i) Adults aged ≥ 18 years with ESKD, (ii) receiving stable maintenance HD for at least three months with a thrice-weekly dialysis schedule, with (iii) prescription of either SHF-HD or post-dilution OL-HDF, and (iv) use of an arteriovenous fistula, arteriovenous graft, or tunneled cuffed catheter as vascular access.

Exclusion criteria were (i) Inadequate blood flow rate (< 250 mL/min for OL-HDF or < 200 mL/min for SHF-HD), (ii) Residual urine volume > 100 mL/24 hours, and (iii) Presence of severe acute illness.

Sample Size

Because this study was designed as a cross-sectional, exploratory analysis conducted within a fixed period and a defined dialysis population, no formal sample size calculation was performed. The number of participants was determined by the availability of eligible patients meeting the inclusion criteria. Convenience sampling was applied among patients who met the criteria and consented within the study timeframe. No formal sample size calculation was performed, and the study was not powered to detect small between-group differences.

A total of 30 ESKD patients undergoing HD were included, of whom 15 were treated with SHF-HD, and 15 were treated with OL-HDF. 

Outcomes

The primary outcome was the short-term efficacy of SHF-HD, assessed by the reduction ratio (RR) of middle molecules after dialysis, compared with OL-HDF. Secondary outcomes comprised intradialytic complications and post-dialysis changes in serum albumin and CRP, representing treatment safety and biocompatibility, and were likewise compared with OL-HDF.

Data collection and laboratory measurements

In Vietnam, the indication of OL-HDF and HD using SHF dialyzers follows the criteria established by the Ministry of Health (Official Decision No. 3365/QĐ-BYT) [21]. Patients are eligible for OL-HDF when presenting with any of the following conditions: hyperphosphatemia-related disorders, malnutrition, anemia with poor response to erythropoietin, uremic pruritus, infection-related complications, amyloidosis, cardiovascular or neurological complications related to ESKD, refractory hypertension, or in acute emergencies requiring cytokine removal when conventional hemodialysis is insufficient. The indication of hemodialysis using SHF dialyzers is similar to that for conventional low-flux and high-flux dialyzers. 

At the Dialysis Unit of People’s Hospital 115, treatment modality assignment adheres to both national and institutional policies. Patients are prescribed OL-HDF if they meet the national criteria. The use of SHF dialyzer is indicated for patients who meet the criteria for OL-HDF but are unable to undergo the procedure due to specific limitations such as: patient’s refusal, financial constraints, poor vascular access, or failure to achieve the required Qb for HDF. The decision to use the SHF dialyzer, therefore, represents a technical and pragmatic institution aimed at enhancing the middle molecule clearance in patients who cannot access OL-HDF, while maintaining the same clinical indication framework defined by the Vietnam Ministry of Health.

Dialysis was performed using Fresenius dialysis machines (Fresenius Medical Care AG, Bad Homburg, Germany), model 4008S for SHF-HD and 5008S for OL-HDF, both of which were connected to a two-stage reverse osmosis system with DIASAFE®plus filters (Fresenius Medical Care AG) to ensure water purity. SHF sessions used Elisio-17HX or Elisio-19HX dialyzers (Nipro Corporation, Osaka, Japan). OL-HDF was delivered in post-dilution mode. All treatments used a dialysate flow rate of 500 mL/minute and a treatment duration (dialysis session) of 240 minutes. All participants received bicarbonate-based dialysate, and dialyzers were single-use during sampling to eliminate reuse bias. 

For each patient, a venous blood sample was drawn immediately before and after a single 240-minute dialysis session using a slow-flow technique following the 2015 Kidney Disease Outcomes Quality Initiative (KDOQI) guideline [2] to prevent access-related fluctuations. Main biochemical molecules included B2M, interleukin-6 (IL6), parathyroid hormone (PTH), leptin, albumin, and CRP. Although serum albumin was measured pre- and post-dialysis, albumin concentration in dialysate or ultrafiltrate was not directly quantified.

The corrected reduction ratio was calculated following the hematocrit-corrected formula according to Schneditz et al. [22], applied for PTH, β2MG, IL6, and Leptin. The reduction ratio of Urea and Phosphate was calculated using the normal formula. 

Statistical analysis

All data were analyzed using R Console v4.5.1 (Posit PBC, Boston, Massachusetts, United States), Microsoft Excel (Microsoft Corporation, Redmond, Washington, United States), and Word 2021 (Microsoft Corporation) for data handling and reporting. Data normality was evaluated with the Shapiro-Wilk test. Continuous variables were presented as mean ± SD if normally distributed, or median (interquartile range (IQR)) otherwise. Independent samples were compared using Welch’s t-test or Mann-Whitney U test, while paired data were analyzed with the paired t-test or Wilcoxon signed-rank test. p-values below 0.05 were considered statistically significant. 

## Results

Baseline characteristics

A total of 30 ESKD patients undergoing maintenance HD who met the study criteria were enrolled. Of these, 15 patients were treated with SHF-HD (SHF group) and 15 patients received OL-HDF (HDF group). Across the study population, 46.8% were male, and the median age was 48.0 (IQR, 39.0-58.9) years. Blood pressure, BMI, and dialysis vintage have no statistically significant differences between the two groups. Hypertension and diabetes mellitus were the two most common comorbidities, accounting for 73.3% and 53.5% of all participants, respectively. Further details are provided in Table 1. 

**Table 1:** Characteristic of study population Groups were compared using the Mann–Whitney U test or chi-square/Fisher’s exact test, as appropriate; p < 0.05 was considered statistically significant. HDF, online hemodiafiltration; SHF, super high-flux hemodialysis; BMI, body mass index; BP, blood pressure; AVF, arteriovenous fistula; CVD, cardiovascular disease.

Characteristic	Total (n = 30)	HDF (n = 15)	SHF (n = 15)	p-value
Male, n (%)	14 (46.7)	8 (53.5)	6 (40.0)	—
Age (years), median (IQR)	48.0 (39.0-58.9)	39.0 (36.5-51.5)	54.0 (45.0-61.0)	0.054
					BMI (kg/m²), median (IQR)	22.5 (19.3-24.8)	22.8 (21.7-24.2)	19.8 (18.1-24.7)	0.290
Systolic BP (mmHg), median (IQR)	125.0 (120.0-140.0)	120.0 (110.0-135.0)	130.0 (120.0-140.0)	0.169					
Diastolic BP (mmHg), median (IQR)	70.0 (62.5-80.0)	70.0 (60.0-80.0)	70.0 (70.0-80.0)	0.468
Dialysis vintage (months), median (IQR)	37.0 (29.8-49.8)	43.2 (30.5-61.0)	37.0 [30.5-43.5)	0.253
AVF as vascular access, n (%)	29 (96.7)	15 (100)	14 (93.3)	—
History of kidney transplantation, n (%)	1 (3.3)	1 (6.7)	0 (0.0)	—
Hypertension, n (%)	22 (73.3)	11 (73.3)	11 (73.3)	—
Diabetes, n (%)	16 (53.3)	7 (46.7)	9 (60.0)	—
CVD, n (%)	1 (3.3)	1 (6.7)	0 (0.0)	—

Dialysis parameters and baseline biochemical markers

The Qb in the HDF group was significantly higher than that in the SHF group (p = 0.008). Other treatment session parameters were similar between the two groups. At the baseline, there were no statistically significant differences in any laboratory marker concentrations between the HDF group and the SHF group. Details of all biochemical marker concentrations and dialysis parameters are presented in Table 2. 

**Table 2:** Basline dialysis and laboratory parameters of study population Comparisons between HDF and SHF were performed using the independent samples t-test for normally distributed variables, and the Mann–Whitney U test for non-normally distributed variables; p < 0.05 was considered statistically significant. HDF, online hemodiafiltration; SHF, super high-flux hemodialysis; Qb, blood flow rate; UF, ultrafiltration; Kt/V, dialysis adequacy index; Hb, hemoglobin; CRP, C-reactive protein; Ca, calcium; P, phosphorus; PTH, parathyroid hormone; β2M, beta-2 microglobulin; IL-6, interleukin-6

Parameter	HDF (n=15)	SHF (n=15)	p-value
Blood flow rate (Qb, mL/minute), mean ± SD	270.00 ± 22.04	240.00 ± 23.60	0.008
Substitution flow rate (mL/minute), mean ± SD	71.60 ± 5.81	—	—
Convective volume (L/session), mean ± SD	17.20 ± 1.44	—	—
UF volume (L), median (IQR])	3.00 (3.00–4.00)	4.00 (2.75–4.00)	0.692
Kt/V, median (IQR)	1.43 (1.33–1.60)	1.37 (1.34–1.48)	0.836
Hemoglobin (Hb, g/dL), mean ± SD	10.78 ± 1.60	10.72 ± 1.22	0.909
Urea (mmol/L), mean ± SD	30.82 ± 7.60	34.11 ± 7.52	0.243
Creatinine (µmol/L), mean ± SD	1123.11 ± 264.01	1209.02 ± 308.95	0.420
Albumin (g/L), mean ± SD	36.13 ± 2.04	37.18 ± 1.59	0.128
CRP (mg/L), mean ± SD	10.86 ± 2.52	11.06 ± 3.13	0.843
Calcium (Ca, mmol/L), median (IQR)	2.5 (2.2-2.5)	2.4 (2.2-2.6)	0.917
Phospho (P, mmol/L), median (IQR)	2.50 (2.2-2.8)	2.11 (1.9-2.4)	0.078
PTH (pg/mL), median (IQR)	1.0 (0.6-1.5)	1.1 (0.7-1.8)	0.431
B2M (mg/L), median (IQR)	23.2 (22.1-25.5)	22.9 (21.8-25.7)	0.967
IL6 (pg/mL), median ([IQR)	10.3 (7.3-11.8)	9.2 (6.3-10.4)	0.340
Leptin (ng/mL), median (IQR)	14.3 (3.8-22.5)	5.9 (3.2-16.4)	0.443

Dialysis efficacy

**Figure 1:**
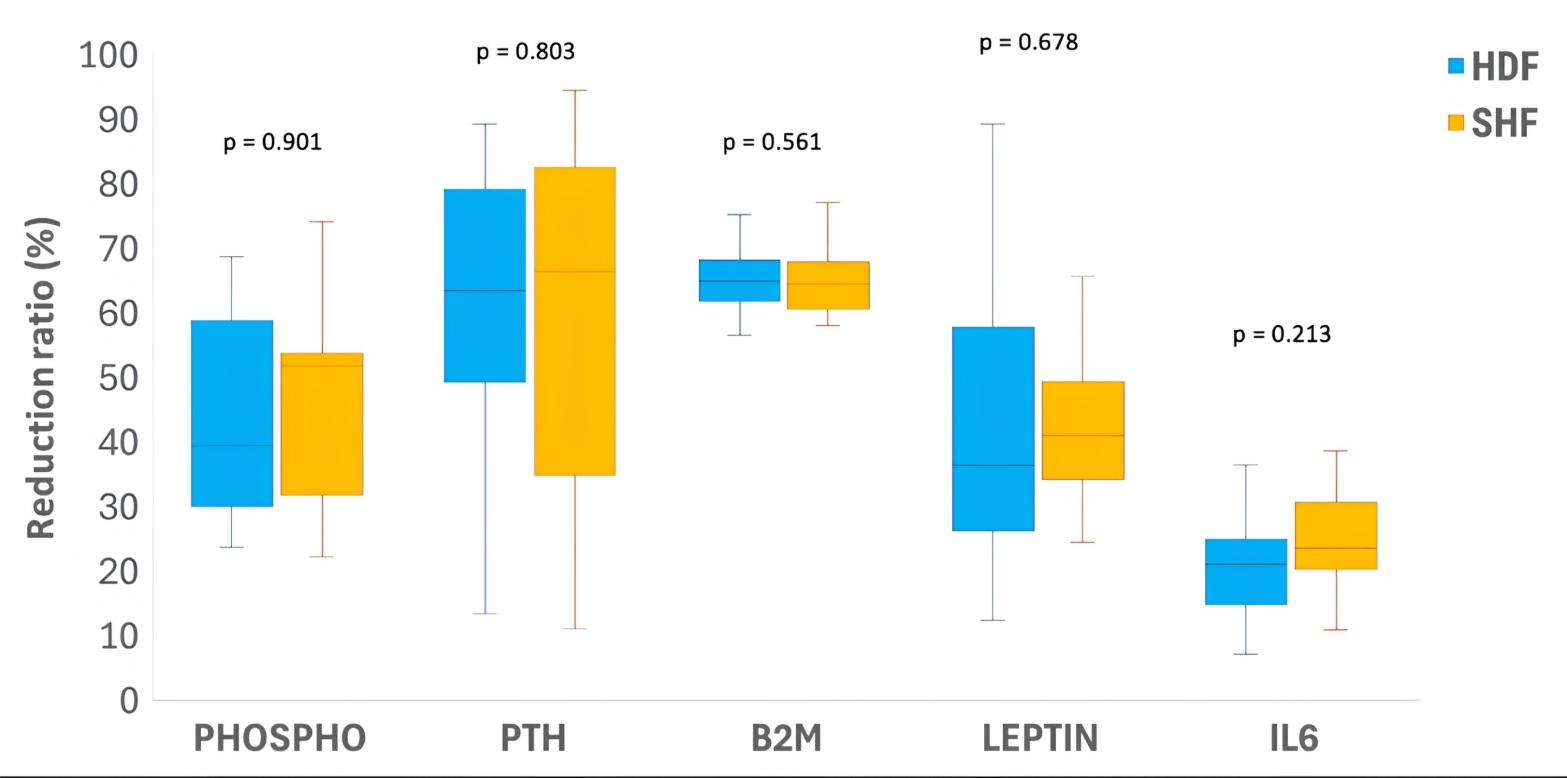
Comparison of solutes clearance between SHF group and HDF group Comparisons between HDF and SHF were performed using the Mann–Whitney U test; p < 0.05 was considered statistically significant. Exact p-values are shown above each comparison. HDF, online hemodiafiltration; SHF, super high-flux hemodialysis; Phospho, phosphorus; PTH, parathyroid hormone; β2M (B2M), beta-2 microglobulin; IL-6, interleukin-6

Regarding the clearance of middle-molecular-weight uremic toxins and cytokines after a single dialysis session, no statistically significant differences were detected between modalities in reduction ratios during the evaluated session. The median RRs of all evaluated middle molecules were not significantly different in the SHF group compared with the HDF group, including phospho (51.71%, IQR 31.91-53.75 vs. 39.52%, IQR, 30.02-58.86; p = 0.901), PTH (66.32%, IQR, 34.87-82.56 vs. 63.57%, IQR, 49.41-79.22; p = 0.803), B2M (65.05%, IQR, 61.61-68.39 vs. 68.20%, IQR, 63.86-67.68, p = 0.561), leptin (41.08%, IQR, 34.25-49.39 vs. 36.36%, IQR, 34.25-49.39, p = 0.678), and IL6 (23.62%, IQR, 20.26-30.72 vs. 21.20%, IQR, 14.76-24.90, p = 0.213), as illustrated in Figure 1.

Safety profile 

In the SHF group, the mean change in serum albumin and CRP after treatment was -0.40 ± 0.68 g/L and -0.48 ± 2.63 mg/L, respectively. In comparison to the HDF group, there were no significant differences in either albumin (-0.44 ± 1.32 g/L, p = 0.911) or CRP (0.16 ± 2.32 mg/L, p = 0.356). 

Throughout all 30 treatment sessions, there were no intradialytic complications in either patient group, including intradialytic hemodynamic instability, cardiac arrhythmias, dialyzer allergic reactions, dialysis disequilibrium syndrome, gastrointestinal syndromes, muscle cramps, and dialyzer rupture. 

## Discussion

Existing evidence on SHF or expanded HD largely originates from high-income countries (Japan, Europe) [12-16] or upper-middle-income settings such as Thailand [9-11]. To our knowledge, this is the first exploratory Vietnamese evaluation comparing SHF-HD with OL-HDF. In this single-session analysis, no statistically significant differences were detected in middle-molecule reduction ratios or short-term safety parameters between groups. These findings indicate similar short-term biochemical performance under the specific treatment conditions studied; however, they do not establish therapeutic equivalence. The primary endpoint, B2M, cytokines, and peptide hormones reduction did not differ significantly between modalities, as expected in four-hour treatments with both groups. Albumin declined insignificantly following SHF-HD and nonsignificantly to OL-HDF, and CRP did not alter in either group. None of the acute safety events were observed. Our findings are in line with prior controlled studies in which SHF-HD achieved comparable clearance of solutes and middle molecules to high-volume OL-HDF, without acute safety concerns [9,11,12,16]. 

B2M is a surrogate for middle molecules and is the basis for dialyzer flux classification in Japan. Over 90% of Japanese HD patients have been treated with type IV/V high-performance membranes for over a decade, which has been associated with lower B2M levels and a decline in amyloidosis complications [13]. Notably, a 2024 Japanese Society for Dialysis Therapy registry study found that the use of type V dialyzers with B2M clearance ≥ 70 mL/min was independently associated with improved two-year survival, with adjusted HR 0.86 (95% CI, 0.80-0.92, p < 0.0001) vs. standard HF membranes [13]. Recent studies in Thailand demonstrated that thrice-weekly SHF-HD eliminates middle molecules and protein-bound toxins as effectively as post-dilution OL-HDF. In one such trial, RRs for indoxyl sulfate and B2M in the SHF-HD group were statistically indistinguishable from those on high-volume OL-HDF, with the convective volume achieved 24-26 L per session [9]. We also measured IL6 and leptin as markers of larger uremic toxins. Both showed a 20-40% reduction per session with no differences between modalities. These larger cytokines and adipokines are poorly removed by diffusion alone, but HDF can remove some through convection. The efficacy is probably supported by the membrane properties of SHF dialyzers. Larger and more uniformly distributed pores increase the sieving coefficient for middle molecules while maintaining a cutoff below albumin’s molecular size. In the dialyzer, the secondary flows and Taylor dispersion have contributed to the amplification of the mass transfer beyond pure diffusion at higher rates of blood flow, which is sometimes called internal convection. All these phenomena, along with the long fibres and the optimised packing of fibres, can increase the transport of larger solutes, which can be transported without the presence of a substitution fluid supplied externally. Some studies even suggest that “expanded HD” with novel membranes might exceed OL-HDF in removing the largest solutes: for example, certain SHF or MCO dialyzers have shown higher clearance of cytokines than OL-HDF [11].

Another important dimension is biocompatibility and inflammation. Persistent low-grade inflammation in dialysis patients has long been tied to their high burden of cardiovascular disease. OL-HDF might attenuate this inflammation over time by two mechanisms: the requirement for ultrapure dialysate reduces exposure to endotoxins, and convective transport can remove some cytokines [3-5]. In the short term, however, we observed no significant difference in inflammatory markers between SHF-HD and OL-HDF. This suggests that both modalities were similarly biocompatible in our setting with real-world dialysis fluid quality in both arms and modern synthetic membranes. Panichi et al. observed that switching from standard HD to HDF led to significant reductions in CRP and IL6 levels over four months, whereas reverting to conventional HD caused these markers to rise again [23]. In our comparison, we did not detect any short-term difference in inflammatory markers between SHF-HD and OL-HDF, which is reasonable given that acute inflammatory shifts rarely emerge within a single session. Even so, membrane design may matter more than the modality alone. Recent work with vitamin E-coated SHF dialyzers is a good example. In a randomized study, the vitamin E-coated membrane achieved middle-molecule clearance similar to an MCO dialyzer, yet it also lowered oxidative and inflammatory biomarkers [24]. Findings like these hint that incremental refinements in membrane biocompatibility might influence the inflammatory response, although whether these effects persist or translate into clinical benefit remains uncertain. Across the 30 sessions we followed, no intradialytic complications occurred, not even the small issues that sometimes surface when membranes are changed. This pattern is similar to the experience reported by Tiranathanagul and colleagues in Thailand, where patients tolerated SHF-HD just as well as OL-HDF and showed no rise in hypotension or other symptoms over their eight-week crossover periods [10]. Taken together, these findings support the view that well-designed high-flux membranes can offer HDF-like clearance while remaining safe and comfortable for patients in the short term, although longer-term effects still need clearer evidence.

Our study has several limitations. The most obvious is the small sample. With only 15 patients in each group, our ability to detect very small differences was inevitably limited. Even so, the overlap in almost every measured parameter was striking, and that consistency gives at least some confidence that the patterns we observed were not simply noise. Another limitation involves the blood-flow rates. Both groups operated at lower Qb than what is typically reported in larger trials [9,11,16], sometimes because of protocol constraints and sometimes because of patient factors that restricted achievable flow. This makes comparison with other studies a little tricky, and it raises the possibility that some performance differences could be masked by lower baseline flow. Lower blood-flow rates often limit the convective volume that HDF can generate, and this can blunt the clinical advantages it would otherwise offer. That concern was relevant in our setting. Although the HDF group in our study operated at a higher Qb than the SHF group, about 270 compared with 240 mL per minute, the SHF treatments still matched HDF in terms of toxin removal. This outcome is striking because, at least in theory, the lower Qb should have placed SHF-HD at a disadvantage. Yet we did not see any meaningful reduction in clearance. It raises the possibility that membrane performance in SHF-HD may compensate for modest flow limitations, although we cannot say how far this effect extends beyond the short-term observations we captured. 

Third, our study did not measure albumin concentration in the dialysate or ultrafiltrate, so we could not directly quantify albumin loss through the dialyzer. We did observe that serum albumin levels after treatment were unchanged in both groups, which is an encouraging indirect indicator that albumin loss was minimal. Furthermore, the mean albumin decrease during SHF-HD (-0.40 g/L) was virtually identical to that during OL-HDF (-0.44 g/L). In OL-HDF, a small amount of albumin, typically 1-3 g per session, is known to be lost into the discarded ultrafiltration fluid. It stands to reason that SHF dialyzers might allow a similar scale of loss. Evidence from other studies supports this: a crossover trial by Tiranathanagul et al. noted slightly higher albumin leakage into dialysate with SHF-HD compared to HDF, but importantly, no significant drop in patients’ serum albumin over 8 weeks of treatment [10]. Likewise, long-term observational work from Thailand found that 15 months on SHF-HD did not lead to hypoalbuminemia; mean serum albumin remained approximately 4.0 g/dL, unchanged from baseline [9]. Still, we acknowledge that without direct measurement, we cannot definitively state the grams of albumin lost in SHF-HD. And finally, because our work was limited to a short observational window, we were not able to examine outcomes that unfold slowly, such as survival, cardiovascular events, or changes in quality of life. That is a real constraint, and it leaves open questions that a longer study would be better suited to answer. Even so, it does not undermine the central pattern we observed: over the short term, SHF-HD performed on par with OL-HDF in both solute clearance and safety. If anything, these limitations simply point toward the next steps, inviting larger and longer studies to determine whether the short-term equivalence we found holds up when patients are followed over months or years.

These results are best interpreted within the context of routine dialysis practice in settings where OL-HDF is not yet widely available, as is still the case in many centers in Vietnam. Conventional high-flux HD remains highly effective for the removal of small solutes, yet its limited capacity for middle-molecule clearance continues to be a concern given the established links between these toxins and complications such as dialysis-related amyloidosis, chronic inflammation, and cardiovascular disease [1]. OL-HDF offers a more efficient solution for this problem, but its broader implementation is constrained by the need for ultrapure water systems, compatible machines, and higher operational costs [19]. Against this background, SHF membranes represent a technically simpler approach that can be readily applied on standard HD platforms and may enhance middle-molecule removal under everyday clinical conditions. From a physiological standpoint, our findings are consistent with the growing body of work supporting the concept of “expanded HD,” in which enhanced diffusive transport and internal convection achieved by SHF or MCO membranes can approach the biochemical clearance profile of post-dilution OL-HDF. Nevertheless, the present study was designed to capture only short-term biochemical and safety outcomes, and any inference beyond acute solute removal would go beyond what our data can reliably support. While SHF-HD may therefore be considered a pragmatic option in centers where OL-HDF cannot be routinely implemented, its influence on longer-term outcomes such as survival, cardiovascular events, or hospitalization cannot be addressed by this study. Importantly, we observed no acute safety concerns, including hemodynamic instability or meaningful albumin loss, which supports the short-term tolerability of SHF-HD in a real-world clinical setting. Whether this favorable short-term biochemical and safety profile translates into sustained clinical benefit will require confirmation in larger studies with longitudinal follow-up.

## Conclusions

In this exploratory, single-session pilot study conducted in routine Vietnamese dialysis practice, SHF-HD demonstrated no statistically significant differences in short-term middle-molecule reduction ratios compared with OL-HDF and was well tolerated acutely. These findings suggest that SHF-HD may provide comparable short-term biochemical performance under moderate blood flow and convective conditions. However, the small sample size, non-randomized allocation, moderate convective volume, and absence of direct albumin quantification preclude conclusions regarding therapeutic equivalence or long-term clinical benefit. Larger prospective studies with longitudinal follow-up are necessary.
